# Supplementary material for: Unveiling the Bioactive Potential of the Invasive Jellyfish Phyllorhiza punctata Through Integrative Transcriptomic and Proteomic Analyses
Source: Biomolecules. 2025 Aug 4;15(8):1121. doi: 10.3390/biom15081121 (PMC12383608; doi:10.3390/biom15081121)
Supplement: Supplementary file 1 [file biomolecules-15-01121-s001.zip › Suplementary Material/FileS3/PEPFOLD4/XP_065052192_5245/clusters.html]

 PEP-FOLD clusters


Download archive of all models  here   

|  |  |  |
| --- | --- | --- |
| # | file | sOPEP |
| # cluster 1 - Size: 33 |  |  |
|  | XP\_065052192\_5245-model1 | -13.539 |
|  | XP\_065052192\_5245-model1.1 | -13.539 |
|  | XP\_065052192\_5245-model1.2 | -13.539 |
|  | XP\_065052192\_5245-model1.3 | -13.539 |
|  | XP\_065052192\_5245-model1.4 | -13.539 |
|  | XP\_065052192\_5245-model1.5 | -13.539 |
|  | XP\_065052192\_5245-model1.6 | -13.539 |
|  | XP\_065052192\_5245-model1.7 | -13.539 |
|  | XP\_065052192\_5245-model1.8 | -13.539 |
|  | XP\_065052192\_5245-model1.9 | -13.539 |
|  | XP\_065052192\_5245-model1.10 | -13.539 |
|  | XP\_065052192\_5245-model1.11 | -13.539 |
|  | XP\_065052192\_5245-model1.12 | -13.539 |
|  | XP\_065052192\_5245-model1.13 | -13.539 |
|  | XP\_065052192\_5245-model1.14 | -13.498 |
|  | XP\_065052192\_5245-model1.15 | -13.498 |
|  | XP\_065052192\_5245-model1.16 | -13.498 |
|  | XP\_065052192\_5245-model1.17 | -13.498 |
|  | XP\_065052192\_5245-model1.18 | -13.498 |
|  | XP\_065052192\_5245-model1.19 | -13.4796 |
|  | XP\_065052192\_5245-model1.20 | -13.4796 |
|  | XP\_065052192\_5245-model1.21 | -13.4796 |
|  | XP\_065052192\_5245-model1.22 | -13.435 |
|  | XP\_065052192\_5245-model1.23 | -13.4325 |
|  | XP\_065052192\_5245-model1.24 | -13.3067 |
|  | XP\_065052192\_5245-model1.25 | -13.3067 |
|  | XP\_065052192\_5245-model1.26 | -13.3067 |
|  | XP\_065052192\_5245-model1.27 | -13.3067 |
|  | XP\_065052192\_5245-model1.28 | -13.3067 |
|  | XP\_065052192\_5245-model1.29 | -13.256 |
|  | XP\_065052192\_5245-model1.30 | -13.2361 |
|  | XP\_065052192\_5245-model1.31 | -13.1633 |
|  | XP\_065052192\_5245-model1.32 | -12.8756 |
| # cluster 2 - Size: 1 |  |  |
|  | XP\_065052192\_5245-model2 | -7.52186 |
| # cluster 3 - Size: 5 |  |  |
|  | XP\_065052192\_5245-model3 | -4.92363 |
|  | XP\_065052192\_5245-model3.1 | -4.65586 |
|  | XP\_065052192\_5245-model3.2 | -4.56374 |
|  | XP\_065052192\_5245-model3.3 | -4.37723 |
|  | XP\_065052192\_5245-model3.4 | -4.13738 |
| # cluster 4 - Size: 8 |  |  |
|  | XP\_065052192\_5245-model4 | -4.74753 |
|  | XP\_065052192\_5245-model4.1 | -4.33217 |
|  | XP\_065052192\_5245-model4.2 | -4.30702 |
|  | XP\_065052192\_5245-model4.3 | -4.2931 |
|  | XP\_065052192\_5245-model4.4 | -4.21236 |
|  | XP\_065052192\_5245-model4.5 | -4.19485 |
|  | XP\_065052192\_5245-model4.6 | -4.09905 |
|  | XP\_065052192\_5245-model4.7 | -4.08304 |
| # cluster 5 - Size: 4 |  |  |
|  | XP\_065052192\_5245-model5 | -4.43249 |
|  | XP\_065052192\_5245-model5.1 | -4.1556 |
|  | XP\_065052192\_5245-model5.2 | -4.1077 |
|  | XP\_065052192\_5245-model5.3 | -4.06136 |
| # cluster 6 - Size: 1 |  |  |
|  | XP\_065052192\_5245-model6 | -4.36785 |
| # cluster 7 - Size: 6 |  |  |
|  | XP\_065052192\_5245-model7 | -4.31705 |
|  | XP\_065052192\_5245-model7.1 | -4.26838 |
|  | XP\_065052192\_5245-model7.2 | -4.15836 |
|  | XP\_065052192\_5245-model7.3 | -4.08707 |
|  | XP\_065052192\_5245-model7.4 | -4.08528 |
|  | XP\_065052192\_5245-model7.5 | -3.96131 |
| # cluster 8 - Size: 1 |  |  |
|  | XP\_065052192\_5245-model8 | -4.30731 |
| # cluster 9 - Size: 9 |  |  |
|  | XP\_065052192\_5245-model9 | -4.28235 |
|  | XP\_065052192\_5245-model9.1 | -4.2189 |
|  | XP\_065052192\_5245-model9.2 | -4.20009 |
|  | XP\_065052192\_5245-model9.3 | -4.15299 |
|  | XP\_065052192\_5245-model9.4 | -4.07498 |
|  | XP\_065052192\_5245-model9.5 | -4.05813 |
|  | XP\_065052192\_5245-model9.6 | -4.02654 |
|  | XP\_065052192\_5245-model9.7 | -4.00521 |
|  | XP\_065052192\_5245-model9.8 | -4.00469 |
| # cluster 10 - Size: 13 |  |  |
|  | XP\_065052192\_5245-model10 | -4.27127 |
|  | XP\_065052192\_5245-model10.1 | -4.10745 |
|  | XP\_065052192\_5245-model10.2 | -4.08395 |
|  | XP\_065052192\_5245-model10.3 | -4.06125 |
|  | XP\_065052192\_5245-model10.4 | -4.04887 |
|  | XP\_065052192\_5245-model10.5 | -4.03427 |
|  | XP\_065052192\_5245-model10.6 | -4.02989 |
|  | XP\_065052192\_5245-model10.7 | -4.01439 |
|  | XP\_065052192\_5245-model10.8 | -3.94853 |
|  | XP\_065052192\_5245-model10.9 | -3.93926 |
|  | XP\_065052192\_5245-model10.10 | -3.92602 |
|  | XP\_065052192\_5245-model10.11 | -3.90586 |
|  | XP\_065052192\_5245-model10.12 | -3.86996 |
| # cluster 11 - Size: 11 |  |  |
|  | XP\_065052192\_5245-model11 | -4.21868 |
|  | XP\_065052192\_5245-model11.1 | -4.20725 |
|  | XP\_065052192\_5245-model11.2 | -4.17502 |
|  | XP\_065052192\_5245-model11.3 | -4.16451 |
|  | XP\_065052192\_5245-model11.4 | -4.10603 |
|  | XP\_065052192\_5245-model11.5 | -4.09877 |
|  | XP\_065052192\_5245-model11.6 | -4.0937 |
|  | XP\_065052192\_5245-model11.7 | -4.01722 |
|  | XP\_065052192\_5245-model11.8 | -3.98753 |
|  | XP\_065052192\_5245-model11.9 | -3.96309 |
|  | XP\_065052192\_5245-model11.10 | -3.88587 |
| # cluster 12 - Size: 1 |  |  |
|  | XP\_065052192\_5245-model12 | -4.17215 |
| # cluster 13 - Size: 1 |  |  |
|  | XP\_065052192\_5245-model13 | -4.12775 |
| # cluster 14 - Size: 4 |  |  |
|  | XP\_065052192\_5245-model14 | -4.12181 |
|  | XP\_065052192\_5245-model14.1 | -4.03451 |
|  | XP\_065052192\_5245-model14.2 | -4.02019 |
|  | XP\_065052192\_5245-model14.3 | -4.002 |
| # cluster 15 - Size: 1 |  |  |
|  | XP\_065052192\_5245-model15 | -3.97008 |
| # cluster 16 - Size: 1 |  |  |
|  | XP\_065052192\_5245-model16 | -3.93802 |
|  |  |  |
